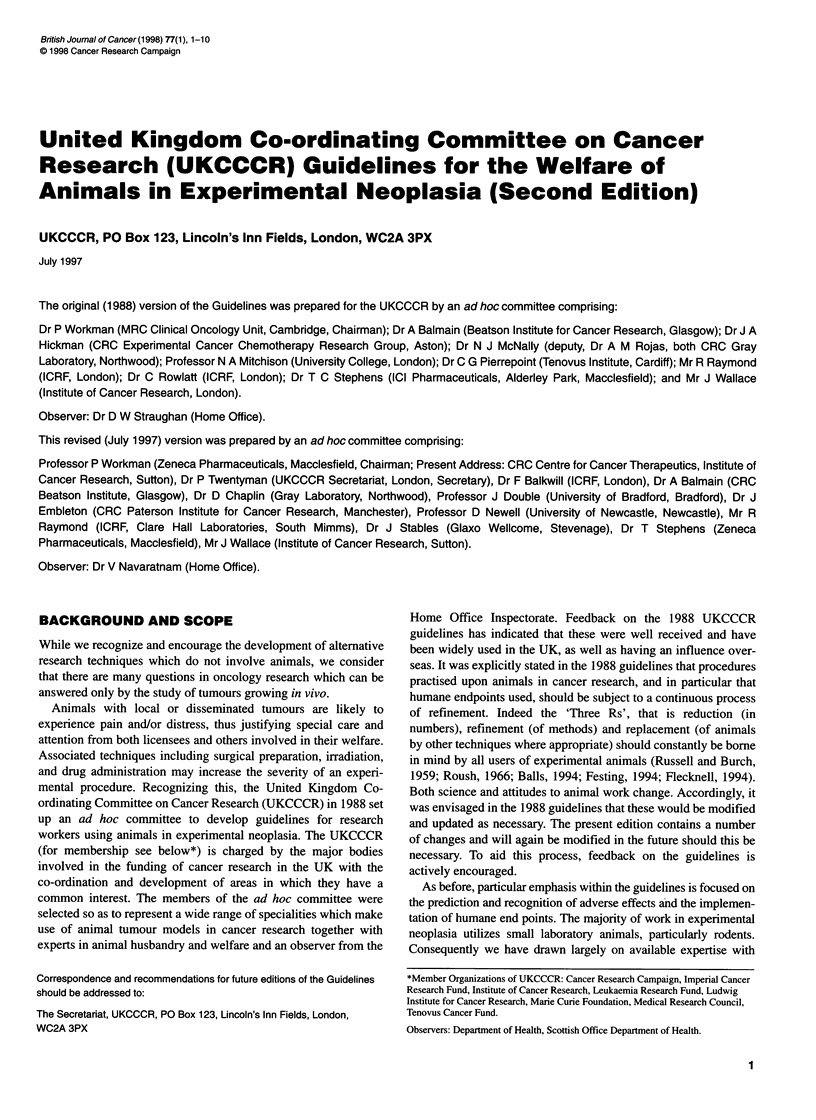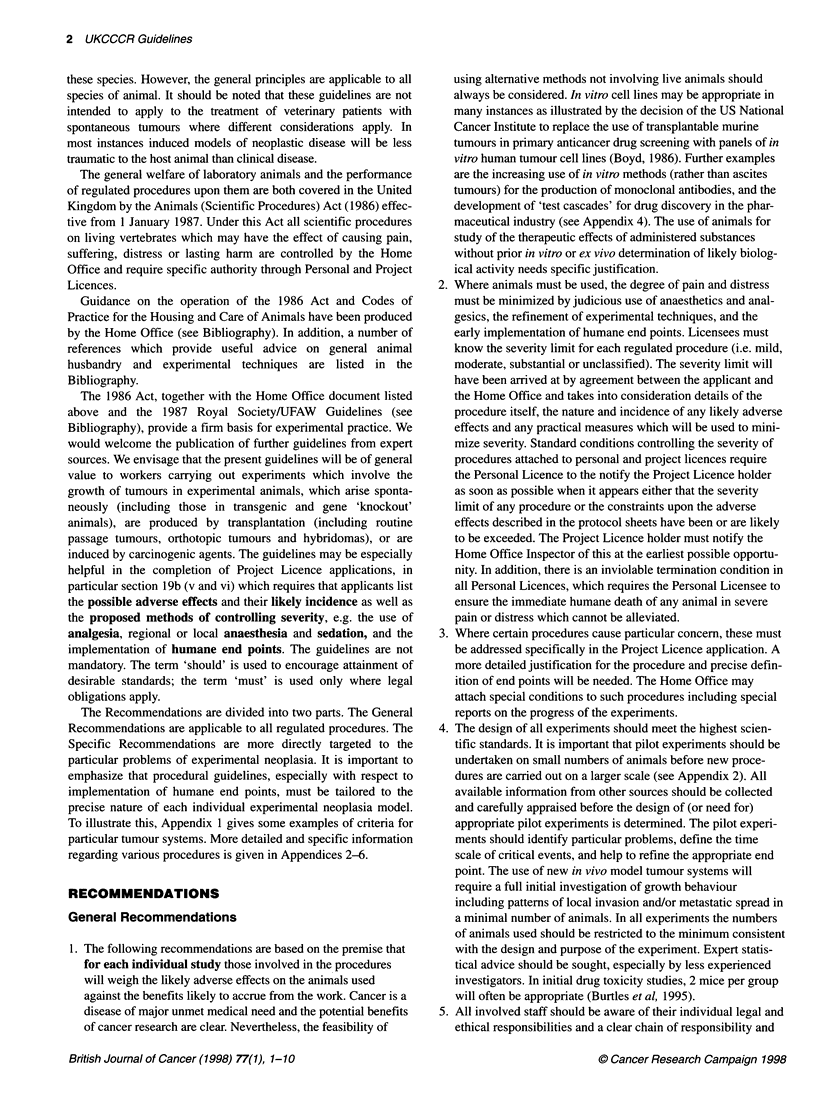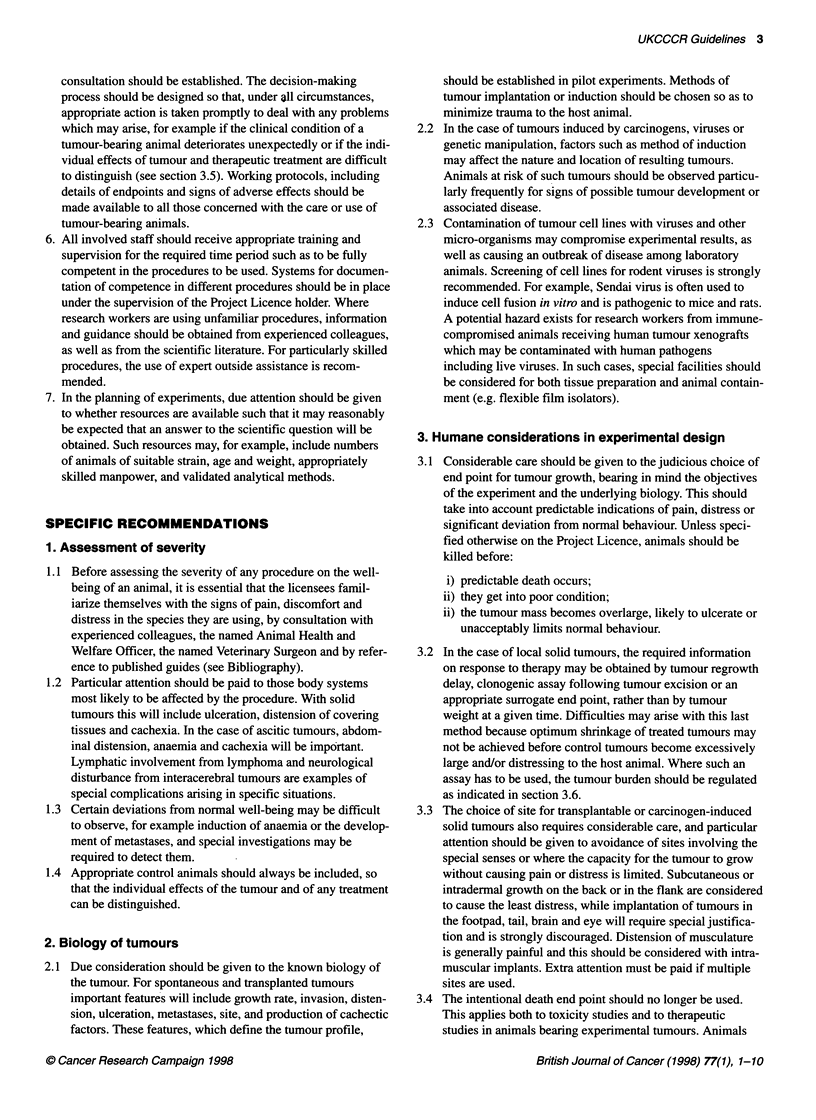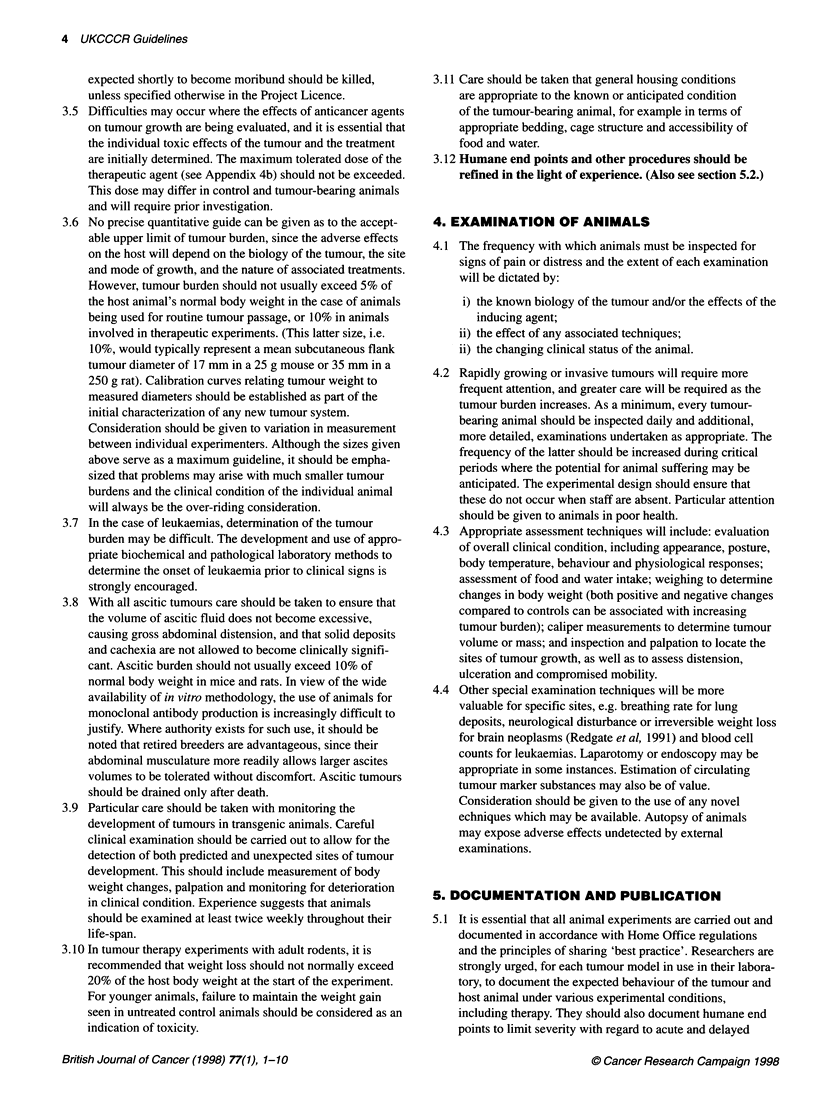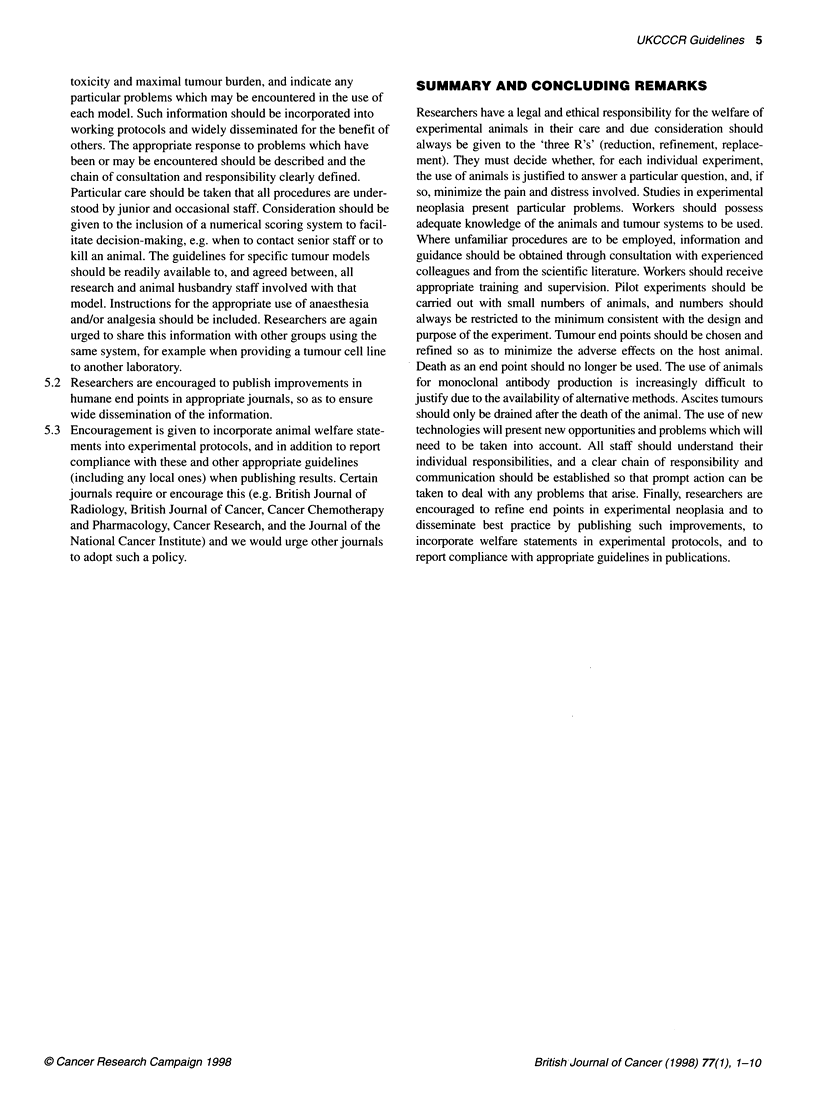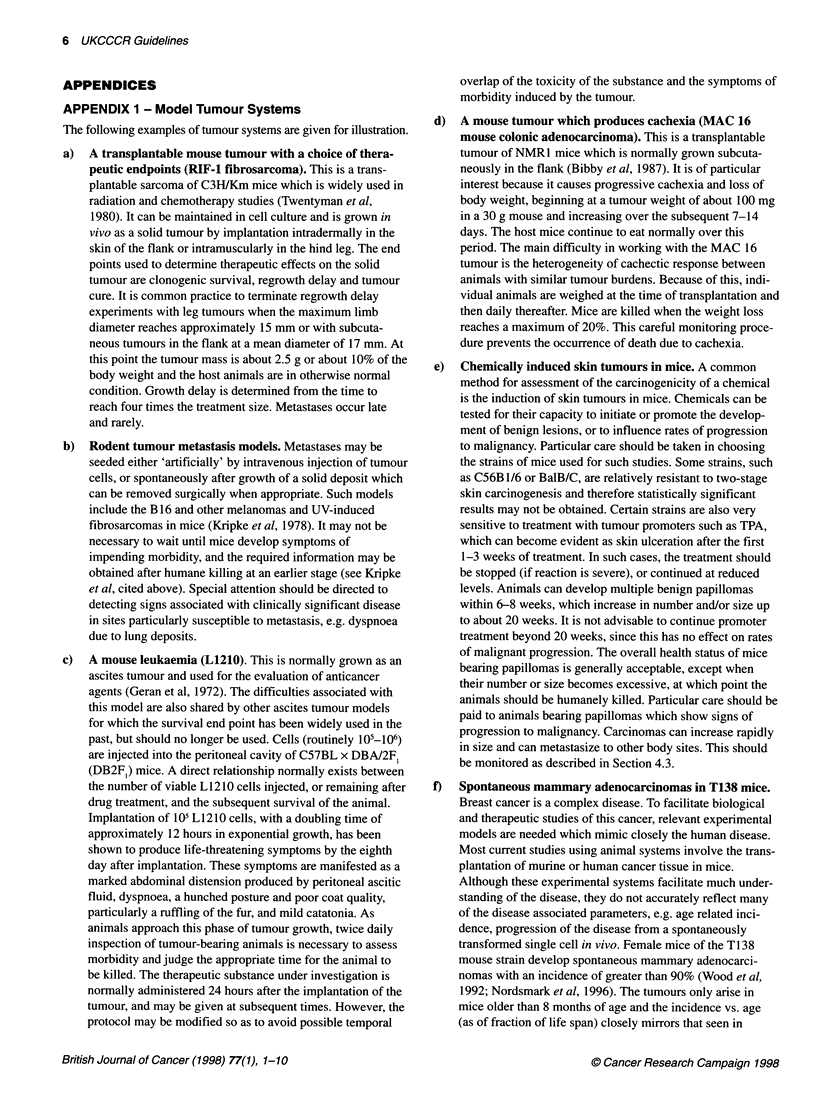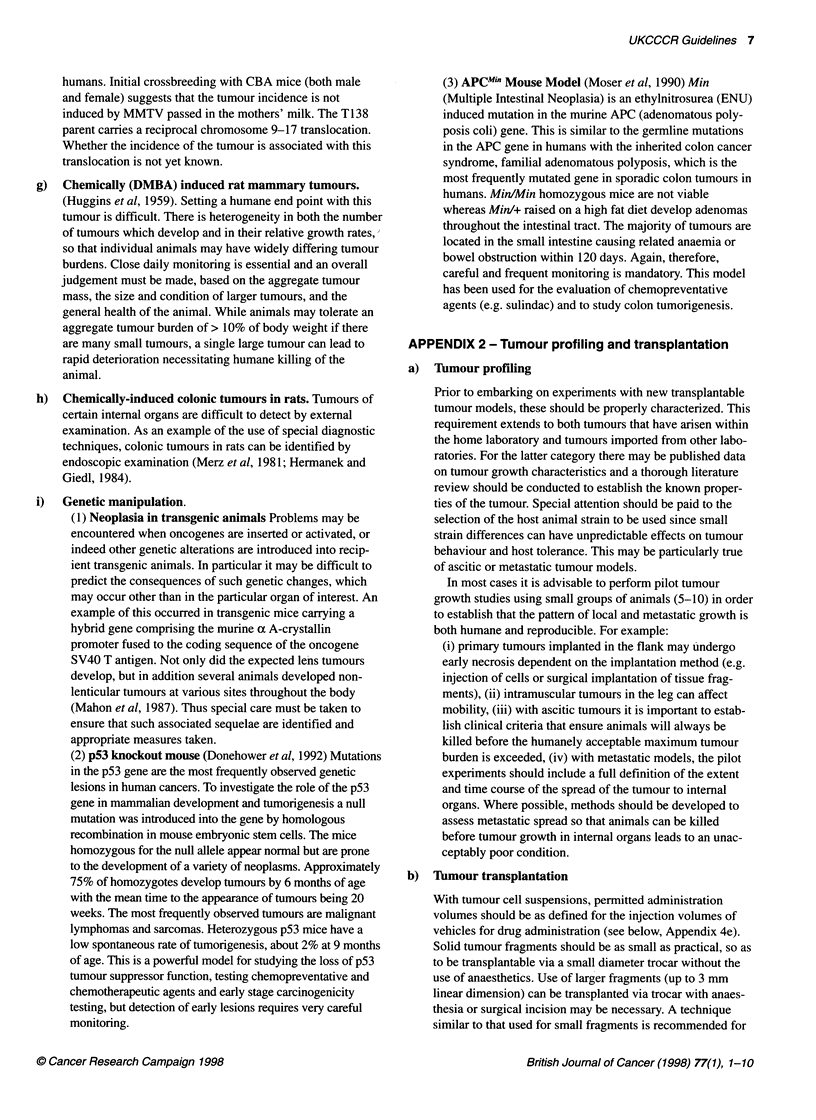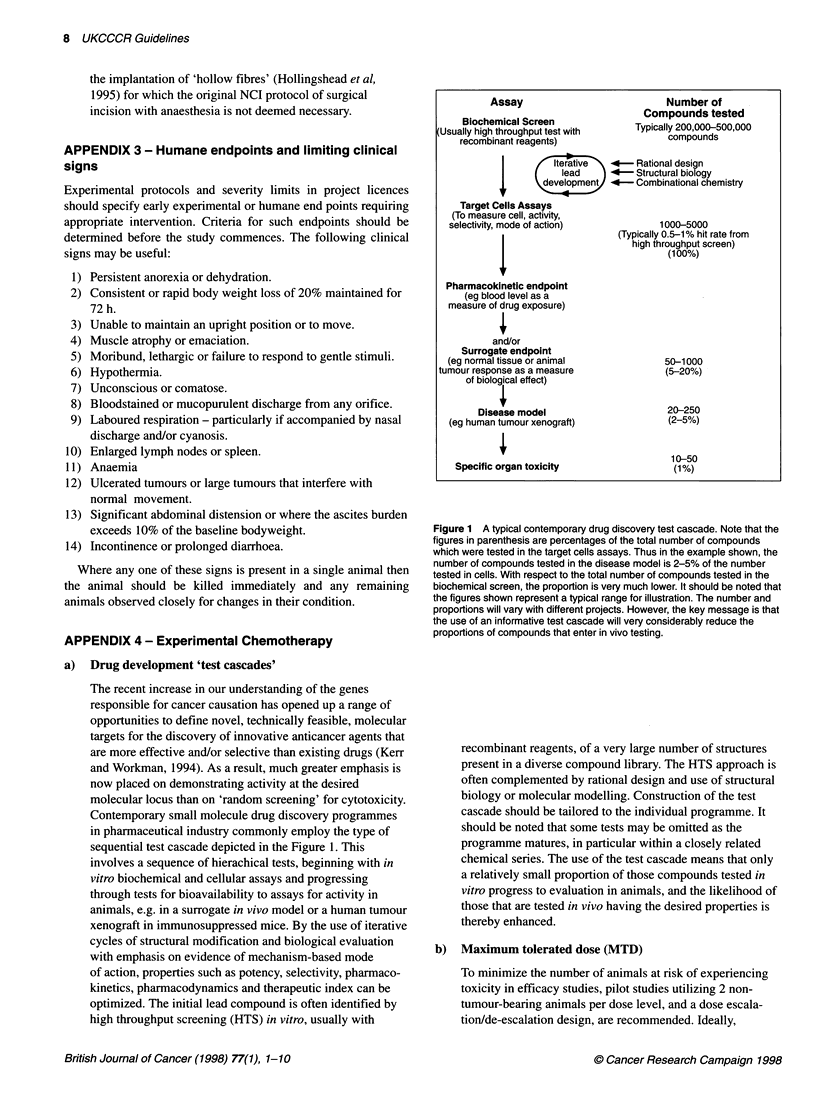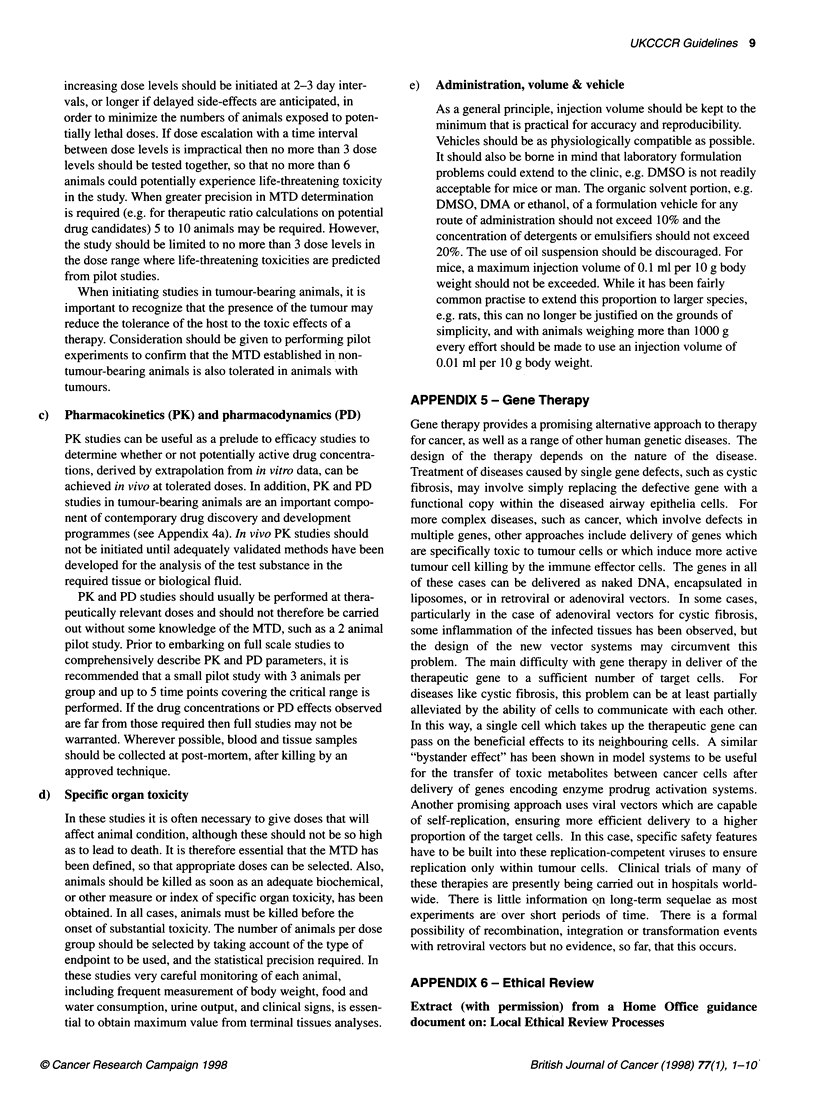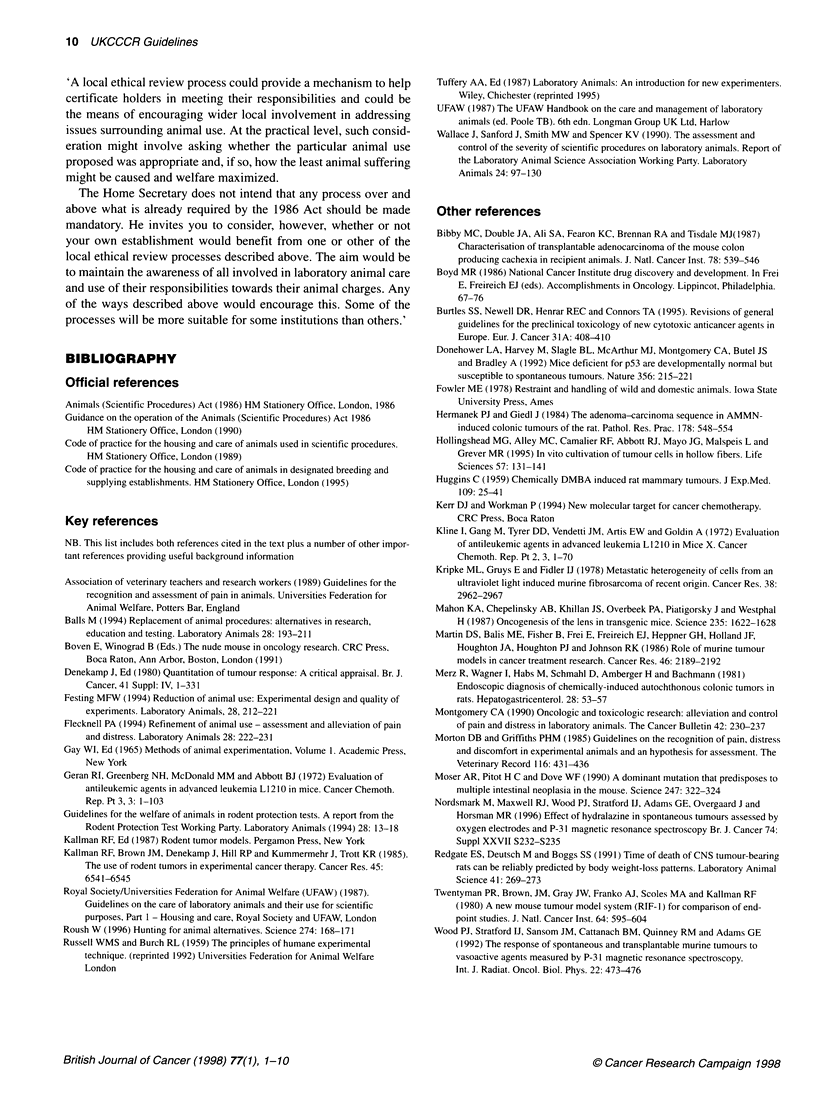# United Kingdom Co-ordinating Committee on Cancer Research (UKCCCR) Guidelines for the Welfare of Animals in Experimental Neoplasia (Second Edition).

**DOI:** 10.1038/bjc.1998.1

**Published:** 1998

**Authors:** 


British Journal of Cancer (1998) 77(1), 1–10
© 1998 Cancer Research Campaign

### United Kingdom Co-ordinating Committee on Cancer Research (UKCCCR) Guidelines for the Welfare of Animals in Experimental Neoplasia (Second Edition)

**UKCCCR, PO Box 123, Lincoln's Inn Fields, London, WC2A 3PX**

July 1997

The original (1988) version of the Guidelines was prepared for the UKCCCR by an *ad hoc* committee comprising:

Dr P Workman (MRC Clinical Oncology Unit, Cambridge, Chairman); Dr A Balmain (Beatson Institute for Cancer Research, Glasgow); Dr J A Hickman (CRC Experimental Cancer Chemotherapy Research Group, Aston); Dr N J McNally (deputy, Dr A M Rojas, both CRC Gray Laboratory, Northwood); Professor N A Mitchison (University College, London); Dr C G Pierrepoint (Tenovus Institute, Cardiff); Mr R Raymond (ICRF, London); Dr C Rowlatt (ICRF, London); Dr T C Stephens (ICI Pharmaceuticals, Alderley Park, Macclesfield); and Mr J Wallace (Institute of Cancer Research, London).

Observer: Dr D W Straughan (Home Office).

This revised (July 1997) version was prepared by an *ad hoc* committee comprising:

Professor P Workman (Zeneca Pharmaceuticals, Macclesfield, Chairman; Present Address: CRC Centre for Cancer Therapeutics, Institute of Cancer Research, Sutton), Dr P Twentyman (UKCCCR Secretariat, London, Secretary), Dr F Balkwill (ICRF, London), Dr A Balmain (CRC Beatson Institute, Glasgow), Dr D Chaplin (Gray Laboratory, Northwood), Professor J Double (University of Bradford, Bradford), Dr J Embleton (CRC Paterson Institute for Cancer Research, Manchester), Professor D Newell (University of Newcastle, Newcastle), Mr R Raymond (ICRF, Clare Hall Laboratories, South Mimms), Dr J Stables (Glaxo Wellcome, Stevenage), Dr T Stephens (Zeneca Pharmaceuticals, Macclesfield), Mr J Wallace (Institute of Cancer Research, Sutton).

Observer: Dr V Navaratnam (Home Office).

### BACKGROUND AND SCOPE

While we recognize and encourage the development of alternative research techniques which do not involve animals, we consider that there are many questions in oncology research which can be answered only by the study of tumours growing *in vivo*.

Animals with local or disseminated tumours are likely to experience pain and/or distress, thus justifying special care and attention from both licensees and others involved in their welfare. Associated techniques including surgical preparation, irradiation, and drug administration may increase the severity of an experimental procedure. Recognizing this, the United Kingdom Co-ordinating Committee on Cancer Research (UKCCCR) in 1988 set up an *ad hoc* committee to develop guidelines for research workers using animals in experimental neoplasia. The UKCCCR (for membership see below*) is charged by the major bodies involved in the funding of cancer research in the UK with the co-ordination and development of areas in which they have a common interest. The members of the *ad hoc* committee were selected so as to represent a wide range of specialities which make use of animal tumour models in cancer research together with experts in animal husbandry and welfare and an observer from the Home Office Inspectorate. Feedback on the 1988 UKCCCR guidelines has indicated that these were well received and have been widely used in the UK, as well as having an influence overseas. It was explicitly stated in the 1988 guidelines that procedures practised upon animals in cancer research, and in particular that humane endpoints used, should be subject to a continuous process of refinement. Indeed the 'Three Rs', that is reduction (in numbers), refinement (of methods) and replacement (of animals by other techniques where appropriate) should constantly be borne in mind by all users of experimental animals (Russell and Burch, 1959; Roush, 1966; Balls, 1994; Festing, 1994; Flecknell, 1994). Both science and attitudes to animal work change. Accordingly, it was envisaged in the 1988 guidelines that these would be modified and updated as necessary. The present edition contains a number of changes and will again be modified in the future should this be necessary. To aid this process, feedback on the guidelines is actively encouraged.

As before, particular emphasis within the guidelines is focused on the prediction and recognition of adverse effects and the implementation of humane end points. The majority of work in experimental neoplasia utilizes small laboratory animals, particularly rodents. Consequently we have drawn largely on available expertise with

Correspondence and recommendations for future editions of the Guidelines should be addressed to:

The Secretariat, UKCCCR, PO Box 123, Lincoln's Inn Fields, London, WC2A 3PX

*Member Organizations of UKCCCR: Cancer Research Campaign, Imperial Cancer Research Fund, Institute of Cancer Research, Leukaemia Research Fund, Ludwig Institute for Cancer Research, Marie Curie Foundation, Medical Research Council, Tenovus Cancer Fund.

Observers: Department of Health, Scottish Office Department of Health.

these species. However, the general principles are applicable to all species of animal. It should be noted that these guidelines are not intended to apply to the treatment of veterinary patients with spontaneous tumours where different considerations apply. In most instances induced models of neoplastic disease will be less traumatic to the host animal than clinical disease.

The general welfare of laboratory animals and the performance of regulated procedures upon them are both covered in the United Kingdom by the Animals (Scientific Procedures) Act (1986) effective from 1 January 1987. Under this Act all scientific procedures on living vertebrates which may have the effect of causing pain, suffering, distress or lasting harm are controlled by the Home Office and require specific authority through Personal and Project Licences.

Guidance on the operation of the 1986 Act and Codes of Practice for the Housing and Care of Animals have been produced by the Home Office (see Bibliography). In addition, a number of references which provide useful advice on general animal husbandry and experimental techniques are listed in the Bibliography.

The 1986 Act, together with the Home Office document listed above and the 1987 Royal Society/UFAW Guidelines (see Bibliography), provide a firm basis for experimental practice. We would welcome the publication of further guidelines from expert sources. We envisage that the present guidelines will be of general value to workers carrying out experiments which involve the growth of tumours in experimental animals, which arise spontaneously (including those in transgenic and gene 'knockout' animals), are produced by transplantation (including routine passage tumours, orthotopic tumours and hybridomas), or are induced by carcinogenic agents. The guidelines may be especially helpful in the completion of Project Licence applications, in particular section 19b (v and vi) which requires that applicants list the **possible adverse effects** and their **likely incidence** as well as the **proposed methods of controlling severity**, e.g. the use of **analgesia**, regional or local **anaesthesia** and **sedation,** and the implementation of **humane end points**. The guidelines are not mandatory. The term 'should' is used to encourage attainment of desirable standards; the term 'must' is used only where legal obligations apply.

The Recommendations are divided into two parts. The General Recommendations are applicable to all regulated procedures. The Specific Recommendations are more directly targeted to the particular problems of experimental neoplasia. It is important to emphasize that procedural guidelines, especially with respect to implementation of humane end points, must be tailored to the precise nature of each individual experimental neoplasia model. To illustrate this, Appendix 1 gives some examples of criteria for particular tumour systems. More detailed and specific information regarding various procedures is given in Appendices 2–6.

### RECOMMENDATIONS

### General Recommendations

1. The following recommendations are based on the premise that **for each individual study** those involved in the procedures will weigh the likely adverse effects on the animals used against the benefits likely to accrue from the work. Cancer is a disease of major unmet medical need and the potential benefits of cancer research are clear. Nevertheless, the feasibility of using alternative methods not involving live animals should always be considered. *In vitro* cell lines may be appropriate in many instances as illustrated by the decision of the US National Cancer Institute to replace the use of transplantable murine tumours in primary anticancer drug screening with panels of *in vitro* human tumour cell lines (Boyd, 1986). Further examples are the increasing use of *in vitro* methods (rather than ascites tumours) for the production of monoclonal antibodies, and the development of 'test cascades' for drug discovery in the pharmaceutical industry (see Appendix 4). The use of animals for study of the therapeutic effects of administered substances without prior *in vitro* or *ex vivo* determination of likely biological activity needs specific justification.
2. Where animals must be used, the degree of pain and distress must be minimized by judicious use of anaesthetics and analgesics, the refinement of experimental techniques, and the early implementation of humane end points. Licensees must know the severity limit for each regulated procedure (i.e. mild, moderate, substantial or unclassified). The severity limit will have been arrived at by agreement between the applicant and the Home Office and takes into consideration details of the procedure itself, the nature and incidence of any likely adverse effects and any practical measures which will be used to minimize severity. Standard conditions controlling the severity of procedures attached to personal and project licences require the Personal Licence to the notify the Project Licence holder as soon as possible when it appears either that the severity limit of any procedure or the constraints upon the adverse effects described in the protocol sheets have been or are likely to be exceeded. The Project Licence holder must notify the Home Office Inspector of this at the earliest possible opportunity. In addition, there is an inviolable termination condition in all Personal Licences, which requires the Personal Licensee to ensure the immediate humane death of any animal in severe pain or distress which cannot be alleviated.
3. Where certain procedures cause particular concern, these must be addressed specifically in the Project Licence application. A more detailed justification for the procedure and precise definition of end points will be needed. The Home Office may attach special conditions to such procedures including special reports on the progress of the experiments.
4. The design of all experiments should meet the highest scientific standards. It is important that pilot experiments should be undertaken on small numbers of animals before new procedures are carried out on a larger scale (see Appendix 2). All available information from other sources should be collected and carefully appraised before the design of (or need for) appropriate pilot experiments is determined. The pilot experiments should identify particular problems, define the time scale of critical events, and help to refine the appropriate end point. The use of new *in vivo* model tumour systems will require a full initial investigation of growth behaviour including patterns of local invasion and/or metastatic spread in a minimal number of animals. In all experiments the numbers of animals used should be restricted to the minimum consistent with the design and purpose of the experiment. Expert statistical advice should be sought, especially by less experienced investigators. In initial drug toxicity studies, 2 mice per group will often be appropriate (Burtles *et al*, 1995).
5. All involved staff should be aware of their individual legal and ethical responsibilities and a clear chain of responsibility and

 *© Cancer Research Campaign 1998*

consultation should be established. The decision-making process should be designed so that, under all circumstances, appropriate action is taken promptly to deal with any problems which may arise, for example if the clinical condition of a tumour-bearing animal deteriorates unexpectedly or if the individual effects of tumour and therapeutic treatment are difficult to distinguish (see section 3.5). Working protocols, including details of endpoints and signs of adverse effects should be made available to all those concerned with the care or use of tumour-bearing animals.

6. All involved staff should receive appropriate training and supervision for the required time period such as to be fully competent in the procedures to be used. Systems for documentation of competence in different procedures should be in place under the supervision of the Project Licence holder. Where research workers are using unfamiliar procedures, information and guidance should be obtained from experienced colleagues, as well as from the scientific literature. For particularly skilled procedures, the use of expert outside assistance is recommended.
7. In the planning of experiments, due attention should be given to whether resources are available such that it may reasonably be expected that an answer to the scientific question will be obtained. Such resources may, for example, include numbers of animals of suitable strain, age and weight, appropriately skilled manpower, and validated analytical methods.

### SPECIFIC RECOMMENDATIONS

### 1. Assessment of severity

1.1 Before assessing the severity of any procedure on the well-being of an animal, it is essential that the licensees familiarize themselves with the signs of pain, discomfort and distress in the species they are using, by consultation with experienced colleagues, the named Animal Health and Welfare Officer, the named Veterinary Surgeon and by reference to published guides (see Bibliography).

1.2 Particular attention should be paid to those body systems most likely to be affected by the procedure. With solid tumours this will include ulceration, distension of covering tissues and cachexia. In the case of ascitic tumours, abdominal distension, anaemia and cachexia will be important. Lymphatic involvement from lymphoma and neurological disturbance from interacerebral tumours are examples of special complications arising in specific situations.

1.3 Certain deviations from normal well-being may be difficult to observe, for example induction of anaemia or the development of metastases, and special investigations may be required to detect them.

1.4 Appropriate control animals should always be included, so that the individual effects of the tumour and of any treatment can be distinguished.

### 2. Biology of tumours

2.1 Due consideration should be given to the known biology of the tumour. For spontaneous and transplanted tumours important features will include growth rate, invasion, distension, ulceration, metastases, site, and production of cachectic factors. These features, which define the tumour profile, should be established in pilot experiments. Methods of tumour implantation or induction should be chosen so as to minimize trauma to the host animal.

2.2 In the case of tumours induced by carcinogens, viruses or genetic manipulation, factors such as method of induction may affect the nature and location of resulting tumours. Animals at risk of such tumours should be observed particularly frequently for signs of possible tumour development or associated disease.

2.3 Contamination of tumour cell lines with viruses and other micro-organisms may compromise experimental results, as well as causing an outbreak of disease among laboratory animals. Screening of cell lines for rodent viruses is strongly recommended. For example, Sendai virus is often used to induce cell fusion *in vitro* and is pathogenic to mice and rats. A potential hazard exists for research workers from immune-compromised animals receiving human tumour xenografts which may be contaminated with human pathogens including live viruses. In such cases, special facilities should be considered for both tissue preparation and animal containment (e.g. flexible film isolators).

### 3. Humane considerations in experimental design

3.1 Considerable care should be given to the judicious choice of end point for tumour growth, bearing in mind the objectives of the experiment and the underlying biology. This should take into account predictable indications of pain, distress or significant deviation from normal behaviour. Unless specified otherwise on the Project Licence, animals should be killed before:

i) predictable death occurs;
ii) they get into poor condition;
ii) the tumour mass becomes overlarge, likely to ulcerate or unacceptably limits normal behaviour.

3.2 In the case of local solid tumours, the required information on response to therapy may be obtained by tumour regrowth delay, clonogenic assay following tumour excision or an appropriate surrogate end point, rather than by tumour weight at a given time. Difficulties may arise with this last method because optimum shrinkage of treated tumours may not be achieved before control tumours become excessively large and/or distressing to the host animal. Where such an assay has to be used, the tumour burden should be regulated as indicated in section 3.6.

3.3 The choice of site for transplantable or carcinogen-induced solid tumours also requires considerable care, and particular attention should be given to avoidance of sites involving the special senses or where the capacity for the tumour to grow without causing pain or distress is limited. Subcutaneous or intradermal growth on the back or in the flank are considered to cause the least distress, while implantation of tumours in the footpad, tail, brain and eye will require special justification and is strongly discouraged. Distension of musculature is generally painful and this should be considered with intramuscular implants. Extra attention must be paid if multiple sites are used.

3.4 The intentional death end point should no longer be used. This applies both to toxicity studies and to therapeutic studies in animals bearing experimental tumours. Animals

© Cancer Research Campaign 1998

expected shortly to become moribund should be killed, unless specified otherwise in the Project Licence.

3.5 Difficulties may occur where the effects of anticancer agents on tumour growth are being evaluated, and it is essential that the individual toxic effects of the tumour and the treatment are initially determined. The maximum tolerated dose of the therapeutic agent (see Appendix 4b) should not be exceeded. This dose may differ in control and tumour-bearing animals and will require prior investigation.

3.6 No precise quantitative guide can be given as to the acceptable upper limit of tumour burden, since the adverse effects on the host will depend on the biology of the tumour, the site and mode of growth, and the nature of associated treatments. However, tumour burden should not usually exceed 5% of the host animal's normal body weight in the case of animals being used for routine tumour passage, or 10% in animals involved in therapeutic experiments. (This latter size, i.e. 10%, would typically represent a mean subcutaneous flank tumour diameter of 17 mm in a 25 g mouse or 35 mm in a 250 g rat). Calibration curves relating tumour weight to measured diameters should be established as part of the initial characterization of any new tumour system. Consideration should be given to variation in measurement between individual experimenters. Although the sizes given above serve as a maximum guideline, it should be emphasized that problems may arise with much smaller tumour burdens and the clinical condition of the individual animal will always be the over-riding consideration.

3.7 In the case of leukaemias, determination of the tumour burden may be difficult. The development and use of appropriate biochemical and pathological laboratory methods to determine the onset of leukaemia prior to clinical signs is strongly encouraged.

3.8 With all ascitic tumours care should be taken to ensure that the volume of ascitic fluid does not become excessive, causing gross abdominal distension, and that solid deposits and cachexia are not allowed to become clinically significant. Ascitic burden should not usually exceed 10% of normal body weight in mice and rats. In view of the wide availability of *in vitro* methodology, the use of animals for monoclonal antibody production is increasingly difficult to justify. Where authority exists for such use, it should be noted that retired breeders are advantageous, since their abdominal musculature more readily allows larger ascites volumes to be tolerated without discomfort. Ascitic tumours should be drained only after death.

3.9 Particular care should be taken with monitoring the development of tumours in transgenic animals. Careful clinical examination should be carried out to allow for the detection of both predicted and unexpected sites of tumour development. This should include measurement of body weight changes, palpation and monitoring for deterioration in clinical condition. Experience suggests that animals should be examined at least twice weekly throughout their life-span.

3.10 In tumour therapy experiments with adult rodents, it is recommended that weight loss should not normally exceed 20% of the host body weight at the start of the experiment. For younger animals, failure to maintain the weight gain seen in untreated control animals should be considered as an indication of toxicity.

3.11 Care should be taken that general housing conditions are appropriate to the known or anticipated condition of the tumour-bearing animal, for example in terms of appropriate bedding, cage structure and accessibility of food and water.

3.12 **Humane end points and other procedures should be refined in the light of experience. (Also see section 5.2.)**

### 4. EXAMINATION OF ANIMALS

4.1 The frequency with which animals must be inspected for signs of pain or distress and the extent of each examination will be dictated by:

i) the known biology of the tumour and/or the effects of the inducing agent;
ii) the effect of any associated techniques;
ii) the changing clinical status of the animal.

4.2 Rapidly growing or invasive tumours will require more frequent attention, and greater care will be required as the tumour burden increases. As a minimum, every tumour-bearing animal should be inspected daily and additional, more detailed, examinations undertaken as appropriate. The frequency of the latter should be increased during critical periods where the potential for animal suffering may be anticipated. The experimental design should ensure that these do not occur when staff are absent. Particular attention should be given to animals in poor health.

4.3 Appropriate assessment techniques will include: evaluation of overall clinical condition, including appearance, posture, body temperature, behaviour and physiological responses; assessment of food and water intake; weighing to determine changes in body weight (both positive and negative changes compared to controls can be associated with increasing tumour burden); caliper measurements to determine tumour volume or mass; and inspection and palpation to locate the sites of tumour growth, as well as to assess distension, ulceration and compromised mobility.

4.4 Other special examination techniques will be more valuable for specific sites, e.g. breathing rate for lung deposits, neurological disturbance or irreversible weight loss for brain neoplasms (Redgate *et al*, 1991) and blood cell counts for leukaemias. Laparotomy or endoscopy may be appropriate in some instances. Estimation of circulating tumour marker substances may also be of value. Consideration should be given to the use of any novel echniques which may be available. Autopsy of animals may expose adverse effects undetected by external examinations.

### 5. DOCUMENTATION AND PUBLICATION

5.1 It is essential that all animal experiments are carried out and documented in accordance with Home Office regulations and the principles of sharing 'best practice'. Researchers are strongly urged, for each tumour model in use in their laboratory, to document the expected behaviour of the tumour and host animal under various experimental conditions, including therapy. They should also document humane end points to limit severity with regard to acute and delayed

© Cancer Research Campaign 1998

toxicity and maximal tumour burden, and indicate any particular problems which may be encountered in the use of each model. Such information should be incorporated into working protocols and widely disseminated for the benefit of others. The appropriate response to problems which have been or may be encountered should be described and the chain of consultation and responsibility clearly defined. Particular care should be taken that all procedures are understood by junior and occasional staff. Consideration should be given to the inclusion of a numerical scoring system to facilitate decision-making, e.g. when to contact senior staff or to kill an animal. The guidelines for specific tumour models should be readily available to, and agreed between, all research and animal husbandry staff involved with that model. Instructions for the appropriate use of anaesthesia and/or analgesia should be included. Researchers are again urged to share this information with other groups using the same system, for example when providing a tumour cell line to another laboratory.

5.2 Researchers are encouraged to publish improvements in humane end points in appropriate journals, so as to ensure wide dissemination of the information.

5.3 Encouragement is given to incorporate animal welfare statements into experimental protocols, and in addition to report compliance with these and other appropriate guidelines (including any local ones) when publishing results. Certain journals require or encourage this (e.g. British Journal of Radiology, British Journal of Cancer, Cancer Chemotherapy and Pharmacology, Cancer Research, and the Journal of the National Cancer Institute) and we would urge other journals to adopt such a policy.

### SUMMARY AND CONCLUDING REMARKS

Researchers have a legal and ethical responsibility for the welfare of experimental animals in their care and due consideration should always be given to the 'three R's' (reduction, refinement, replacement). They must decide whether, for each individual experiment, the use of animals is justified to answer a particular question, and, if so, minimize the pain and distress involved. Studies in experimental neoplasia present particular problems. Workers should possess adequate knowledge of the animals and tumour systems to be used. Where unfamiliar procedures are to be employed, information and guidance should be obtained through consultation with experienced colleagues and from the scientific literature. Workers should receive appropriate training and supervision. Pilot experiments should be carried out with small numbers of animals, and numbers should always be restricted to the minimum consistent with the design and purpose of the experiment. Tumour end points should be chosen and refined so as to minimize the adverse effects on the host animal. Death as an end point should no longer be used. The use of animals for monoclonal antibody production is increasingly difficult to justify due to the availability of alternative methods. Ascites tumours should only be drained after the death of the animal. The use of new technologies will present new opportunities and problems which will need to be taken into account. All staff should understand their individual responsibilities, and a clear chain of responsibility and communication should be established so that prompt action can be taken to deal with any problems that arise. Finally, researchers are encouraged to refine end points in experimental neoplasia and to disseminate best practice by publishing such improvements, to incorporate welfare statements in experimental protocols, and to report compliance with appropriate guidelines in publications.

© Cancer Research Campaign 1998

### APPENDICES

### APPENDIX 1 – Model Tumour Systems

The following examples of tumour systems are given for illustration.

a) **A transplantable mouse tumour with a choice of therapeutic endpoints (RIF-1 fibrosarcoma).** This is a transplantable sarcoma of C3H/Km mice which is widely used in radiation and chemotherapy studies (Twentyman *et al*, 1980). It can be maintained in cell culture and is grown *in vivo* as a solid tumour by implantation intradermally in the skin of the flank or intramuscularly in the hind leg. The end points used to determine therapeutic effects on the solid tumour are clonogenic survival, regrowth delay and tumour cure. It is common practice to terminate regrowth delay experiments with leg tumours when the maximum limb diameter reaches approximately 15 mm or with subcutaneous tumours in the flank at a mean diameter of 17 mm. At this point the tumour mass is about 2.5 g or about 10% of the body weight and the host animals are in otherwise normal condition. Growth delay is determined from the time to reach four times the treatment size. Metastases occur late and rarely.

b) **Rodent tumour metastasis models.** Metastases may be seeded either 'artificially' by intravenous injection of tumour cells, or spontaneously after growth of a solid deposit which can be removed surgically when appropriate. Such models include the B16 and other melanomas and UV-induced fibrosarcomas in mice (Kripke *et al*, 1978). It may not be necessary to wait until mice develop symptoms of impending morbidity, and the required information may be obtained after humane killing at an earlier stage (see Kripke *et al*, cited above). Special attention should be directed to detecting signs associated with clinically significant disease in sites particularly susceptible to metastasis, e.g. dyspnoea due to lung deposits.

c) **A mouse leukaemia (L1210).** This is normally grown as an ascites tumour and used for the evaluation of anticancer agents (Geran et al, 1972). The difficulties associated with this model are also shared by other ascites tumour models for which the survival end point has been widely used in the past, but should no longer be used. Cells (routinely $10^5$–$10^6$) are injected into the peritoneal cavity of C57BL × DBA/2$F_1$ (DB2$F_1$) mice. A direct relationship normally exists between the number of viable L1210 cells injected, or remaining after drug treatment, and the subsequent survival of the animal. Implantation of $10^5$ L1210 cells, with a doubling time of approximately 12 hours in exponential growth, has been shown to produce life-threatening symptoms by the eighth day after implantation. These symptoms are manifested as a marked abdominal distension produced by peritoneal ascitic fluid, dyspnoea, a hunched posture and poor coat quality, particularly a ruffling of the fur, and mild catatonia. As animals approach this phase of tumour growth, twice daily inspection of tumour-bearing animals is necessary to assess morbidity and judge the appropriate time for the animal to be killed. The therapeutic substance under investigation is normally administered 24 hours after the implantation of the tumour, and may be given at subsequent times. However, the protocol may be modified so as to avoid possible temporal overlap of the toxicity of the substance and the symptoms of morbidity induced by the tumour.

d) **A mouse tumour which produces cachexia (MAC 16 mouse colonic adenocarcinoma).** This is a transplantable tumour of NMR1 mice which is normally grown subcutaneously in the flank (Bibby *et al*, 1987). It is of particular interest because it causes progressive cachexia and loss of body weight, beginning at a tumour weight of about 100 mg in a 30 g mouse and increasing over the subsequent 7–14 days. The host mice continue to eat normally over this period. The main difficulty in working with the MAC 16 tumour is the heterogeneity of cachectic response between animals with similar tumour burdens. Because of this, individual animals are weighed at the time of transplantation and then daily thereafter. Mice are killed when the weight loss reaches a maximum of 20%. This careful monitoring procedure prevents the occurrence of death due to cachexia.

e) **Chemically induced skin tumours in mice.** A common method for assessment of the carcinogenicity of a chemical is the induction of skin tumours in mice. Chemicals can be tested for their capacity to initiate or promote the development of benign lesions, or to influence rates of progression to malignancy. Particular care should be taken in choosing the strains of mice used for such studies. Some strains, such as C56B1/6 or BalB/C, are relatively resistant to two-stage skin carcinogenesis and therefore statistically significant results may not be obtained. Certain strains are also very sensitive to treatment with tumour promoters such as TPA, which can become evident as skin ulceration after the first 1–3 weeks of treatment. In such cases, the treatment should be stopped (if reaction is severe), or continued at reduced levels. Animals can develop multiple benign papillomas within 6–8 weeks, which increase in number and/or size up to about 20 weeks. It is not advisable to continue promoter treatment beyond 20 weeks, since this has no effect on rates of malignant progression. The overall health status of mice bearing papillomas is generally acceptable, except when their number or size becomes excessive, at which point the animals should be humanely killed. Particular care should be paid to animals bearing papillomas which show signs of progression to malignancy. Carcinomas can increase rapidly in size and can metastasize to other body sites. This should be monitored as described in Section 4.3.

f) **Spontaneous mammary adenocarcinomas in T138 mice.** Breast cancer is a complex disease. To facilitate biological and therapeutic studies of this cancer, relevant experimental models are needed which mimic closely the human disease. Most current studies using animal systems involve the transplantation of murine or human cancer tissue in mice. Although these experimental systems facilitate much understanding of the disease, they do not accurately reflect many of the disease associated parameters, e.g. age related incidence, progression of the disease from a spontaneously transformed single cell *in vivo*. Female mice of the T138 mouse strain develop spontaneous mammary adenocarcinomas with an incidence of greater than 90% (Wood *et al*, 1992; Nordsmark *et al*, 1996). The tumours only arise in mice older than 8 months of age and the incidence vs. age (as of fraction of life span) closely mirrors that seen in

© Cancer Research Campaign 1998

humans. Initial crossbreeding with CBA mice (both male and female) suggests that the tumour incidence is not induced by MMTV passed in the mothers' milk. The T138 parent carries a reciprocal chromosome 9–17 translocation. Whether the incidence of the tumour is associated with this translocation is not yet known.

g) **Chemically (DMBA) induced rat mammary tumours.** (Huggins *et al*, 1959). Setting a humane end point with this tumour is difficult. There is heterogeneity in both the number of tumours which develop and in their relative growth rates, so that individual animals may have widely differing tumour burdens. Close daily monitoring is essential and an overall judgement must be made, based on the aggregate tumour mass, the size and condition of larger tumours, and the general health of the animal. While animals may tolerate an aggregate tumour burden of > 10% of body weight if there are many small tumours, a single large tumour can lead to rapid deterioration necessitating humane killing of the animal.

h) **Chemically-induced colonic tumours in rats.** Tumours of certain internal organs are difficult to detect by external examination. As an example of the use of special diagnostic techniques, colonic tumours in rats can be identified by endoscopic examination (Merz *et al*, 1981; Hermanek and Giedl, 1984).

i) **Genetic manipulation.**

(1) **Neoplasia in transgenic animals** Problems may be encountered when oncogenes are inserted or activated, or indeed other genetic alterations are introduced into recipient transgenic animals. In particular it may be difficult to predict the consequences of such genetic changes, which may occur other than in the particular organ of interest. An example of this occurred in transgenic mice carrying a hybrid gene comprising the murine α A-crystallin promoter fused to the coding sequence of the oncogene SV40 T antigen. Not only did the expected lens tumours develop, but in addition several animals developed non-lenticular tumours at various sites throughout the body (Mahon *et al*, 1987). Thus special care must be taken to ensure that such associated sequelae are identified and appropriate measures taken.

(2) **p53 knockout mouse** (Donehower *et al*, 1992) Mutations in the p53 gene are the most frequently observed genetic lesions in human cancers. To investigate the role of the p53 gene in mammalian development and tumorigenesis a null mutation was introduced into the gene by homologous recombination in mouse embryonic stem cells. The mice homozygous for the null allele appear normal but are prone to the development of a variety of neoplasms. Approximately 75% of homozygotes develop tumours by 6 months of age with the mean time to the appearance of tumours being 20 weeks. The most frequently observed tumours are malignant lymphomas and sarcomas. Heterozygous p53 mice have a low spontaneous rate of tumorigenesis, about 2% at 9 months of age. This is a powerful model for studying the loss of p53 tumour suppressor function, testing chemopreventative and chemotherapeutic agents and early stage carcinogenicity testing, but detection of early lesions requires very careful monitoring.

(3) **APC$^{Min}$ Mouse Model** (Moser *et al*, 1990) *Min* (Multiple Intestinal Neoplasia) is an ethylnitrosurea (ENU) induced mutation in the murine APC (adenomatous polyposis coli) gene. This is similar to the germline mutations in the APC gene in humans with the inherited colon cancer syndrome, familial adenomatous polyposis, which is the most frequently mutated gene in sporadic colon tumours in humans. *Min/Min* homozygous mice are not viable whereas *Min/+* raised on a high fat diet develop adenomas throughout the intestinal tract. The majority of tumours are located in the small intestine causing related anaemia or bowel obstruction within 120 days. Again, therefore, careful and frequent monitoring is mandatory. This model has been used for the evaluation of chemopreventative agents (e.g. sulindac) and to study colon tumorigenesis.

### APPENDIX 2 – Tumour profiling and transplantation

### a) Tumour profiling

Prior to embarking on experiments with new transplantable tumour models, these should be properly characterized. This requirement extends to both tumours that have arisen within the home laboratory and tumours imported from other laboratories. For the latter category there may be published data on tumour growth characteristics and a thorough literature review should be conducted to establish the known properties of the tumour. Special attention should be paid to the selection of the host animal strain to be used since small strain differences can have unpredictable effects on tumour behaviour and host tolerance. This may be particularly true of ascitic or metastatic tumour models.

In most cases it is advisable to perform pilot tumour growth studies using small groups of animals (5–10) in order to establish that the pattern of local and metastatic growth is both humane and reproducible. For example:

(i) primary tumours implanted in the flank may undergo early necrosis dependent on the implantation method (e.g. injection of cells or surgical implantation of tissue fragments), (ii) intramuscular tumours in the leg can affect mobility, (iii) with ascitic tumours it is important to establish clinical criteria that ensure animals will always be killed before the humanely acceptable maximum tumour burden is exceeded, (iv) with metastatic models, the pilot experiments should include a full definition of the extent and time course of the spread of the tumour to internal organs. Where possible, methods should be developed to assess metastatic spread so that animals can be killed before tumour growth in internal organs leads to an unacceptably poor condition.

### b) Tumour transplantation

With tumour cell suspensions, permitted administration volumes should be defined for the injection volumes of vehicles for drug administration (see below, Appendix 4e). Solid tumour fragments should be as small as practical, so as to be transplantable via a small diameter trocar without the use of anaesthetics. Use of larger fragments (up to 3 mm linear dimension) can be transplanted via trocar with anaesthesia or surgical incision may be necessary. A technique similar to that used for small fragments is recommended for

© Cancer Research Campaign 1998

the implantation of 'hollow fibres' (Hollingshead *et al*, 1995) for which the original NCI protocol of surgical incision with anaesthesia is not deemed necessary.

### APPENDIX 3 – Humane endpoints and limiting clinical signs

Experimental protocols and severity limits in project licences should specify early experimental or humane end points requiring appropriate intervention. Criteria for such endpoints should be determined before the study commences. The following clinical signs may be useful:

1) Persistent anorexia or dehydration.
2) Consistent or rapid body weight loss of 20% maintained for 72 h.
3) Unable to maintain an upright position or to move.
4) Muscle atrophy or emaciation.
5) Moribund, lethargic or failure to respond to gentle stimuli.
6) Hypothermia.
7) Unconscious or comatose.
8) Bloodstained or mucopurulent discharge from any orifice.
9) Laboured respiration – particularly if accompanied by nasal discharge and/or cyanosis.
10) Enlarged lymph nodes or spleen.
11) Anaemia
12) Ulcerated tumours or large tumours that interfere with normal movement.
13) Significant abdominal distension or where the ascites burden exceeds 10% of the baseline bodyweight.
14) Incontinence or prolonged diarrhoea.

Where any one of these signs is present in a single animal then the animal should be killed immediately and any remaining animals observed closely for changes in their condition.

### APPENDIX 4 – Experimental Chemotherapy

### a) Drug development 'test cascades'

The recent increase in our understanding of the genes responsible for cancer causation has opened up a range of opportunities to define novel, technically feasible, molecular targets for the discovery of innovative anticancer agents that are more effective and/or selective than existing drugs (Kerr and Workman, 1994). As a result, much greater emphasis is now placed on demonstrating activity at the desired molecular locus than on 'random screening' for cytotoxicity. Contemporary small molecule drug discovery programmes in pharmaceutical industry commonly employ the type of sequential test cascade depicted in the Figure 1. This involves a sequence of hierachical tests, beginning with *in vitro* biochemical and cellular assays and progressing through tests for bioavailability to assays for activity in animals, e.g. in a surrogate *in vivo* model or a human tumour xenograft in immunosuppressed mice. By the use of iterative cycles of structural modification and biological evaluation with emphasis on evidence of mechanism-based mode of action, properties such as potency, selectivity, pharmacokinetics, pharmacodynamics and therapeutic index can be optimized. The initial lead compound is often identified by high throughput screening (HTS) *in vitro*, usually with

**Figure 1** A typical contemporary drug discovery test cascade. Note that the figures in parenthesis are percentages of the total number of compounds which were tested in the target cells assays. Thus in the example shown, the number of compounds tested in the disease model is 2–5% of the number tested in cells. With respect to the total number of compounds tested in the biochemical screen, the proportion is very much lower. It should be noted that the figures shown represent a typical range for illustration. The number and proportions will vary with different projects. However, the key message is that the use of an informative test cascade will very considerably reduce the proportions of compounds that enter in vivo testing.

recombinant reagents, of a very large number of structures present in a diverse compound library. The HTS approach is often complemented by rational design and use of structural biology or molecular modelling. Construction of the test cascade should be tailored to the individual programme. It should be noted that some tests may be omitted as the programme matures, in particular within a closely related chemical series. The use of the test cascade means that only a relatively small proportion of those compounds tested *in vitro* progress to evaluation in animals, and the likelihood of those that are tested *in vivo* having the desired properties is thereby enhanced.

### b) Maximum tolerated dose (MTD)

To minimize the number of animals at risk of experiencing toxicity in efficacy studies, pilot studies utilizing 2 non-tumour-bearing animals per dose level, and a dose escalation/de-escalation design, are recommended. Ideally,

© Cancer Research Campaign 1998

increasing dose levels should be initiated at 2–3 day intervals, or longer if delayed side-effects are anticipated, in order to minimize the numbers of animals exposed to potentially lethal doses. If dose escalation with a time interval between dose levels is impractical then no more than 3 dose levels should be tested together, so that no more than 6 animals could potentially experience life-threatening toxicity in the study. When greater precision in MTD determination is required (e.g. for therapeutic ratio calculations on potential drug candidates) 5 to 10 animals may be required. However, the study should be limited to no more than 3 dose levels in the dose range where life-threatening toxicities are predicted from pilot studies.

When initiating studies in tumour-bearing animals, it is important to recognize that the presence of the tumour may reduce the tolerance of the host to the toxic effects of a therapy. Consideration should be given to performing pilot experiments to confirm that the MTD established in non-tumour-bearing animals is also tolerated in animals with tumours.

c) **Pharmacokinetics (PK) and pharmacodynamics (PD)**

PK studies can be useful as a prelude to efficacy studies to determine whether or not potentially active drug concentrations, derived by extrapolation from *in vitro* data, can be achieved *in vivo* at tolerated doses. In addition, PK and PD studies in tumour-bearing animals are an important component of contemporary drug discovery and development programmes (see Appendix 4a). *In vivo* PK studies should not be initiated until adequately validated methods have been developed for the analysis of the test substance in the required tissue or biological fluid.

PK and PD studies should usually be performed at therapeutically relevant doses and should not therefore be carried out without some knowledge of the MTD, such as a 2 animal pilot study. Prior to embarking on full scale studies to comprehensively describe PK and PD parameters, it is recommended that a small pilot study with 3 animals per group and up to 5 time points covering the critical range is performed. If the drug concentrations or PD effects observed are far from those required then full studies may not be warranted. Wherever possible, blood and tissue samples should be collected at post-mortem, after killing by an approved technique.

d) **Specific organ toxicity**

In these studies it is often necessary to give doses that will affect animal condition, although these should not be so high as to lead to death. It is therefore essential that the MTD has been defined, so that appropriate doses can be selected. Also, animals should be killed as soon as an adequate biochemical, or other measure or index of specific organ toxicity, has been obtained. In all cases, animals must be killed before the onset of substantial toxicity. The number of animals per dose group should be selected by taking account of the type of endpoint to be used, and the statistical precision required. In these studies very careful monitoring of each animal, including frequent measurement of body weight, food and water consumption, urine output, and clinical signs, is essential to obtain maximum value from terminal tissues analyses.

e) **Administration, volume & vehicle**

As a general principle, injection volume should be kept to the minimum that is practical for accuracy and reproducibility. Vehicles should be as physiologically compatible as possible. It should also be borne in mind that laboratory formulation problems could extend to the clinic, e.g. DMSO is not readily acceptable for mice or man. The organic solvent portion, e.g. DMSO, DMA or ethanol, of a formulation vehicle for any route of administration should not exceed 10% and the concentration of detergents or emulsifiers should not exceed 20%. The use of oil suspension should be discouraged. For mice, a maximum injection volume of 0.1 ml per 10 g body weight should not be exceeded. While it has been fairly common practise to extend this proportion to larger species, e.g. rats, this can no longer be justified on the grounds of simplicity, and with animals weighing more than 1000 g every effort should be made to use an injection volume of 0.01 ml per 10 g body weight.

### APPENDIX 5 – Gene Therapy

Gene therapy provides a promising alternative approach to therapy for cancer, as well as a range of other human genetic diseases. The design of the therapy depends on the nature of the disease. Treatment of diseases caused by single gene defects, such as cystic fibrosis, may involve simply replacing the defective gene with a functional copy within the diseased airway epithelia cells. For more complex diseases, such as cancer, which involve defects in multiple genes, other approaches include delivery of genes which are specifically toxic to tumour cells or which induce more active tumour cell killing by the immune effector cells. The genes in all of these cases can be delivered as naked DNA, encapsulated in liposomes, or in retroviral or adenoviral vectors. In some cases, particularly in the case of adenoviral vectors for cystic fibrosis, some inflammation of the infected tissues has been observed, but the design of the new vector systems may circumvent this problem. The main difficulty with gene therapy in deliver of the therapeutic gene to a sufficient number of target cells. For diseases like cystic fibrosis, this problem can be at least partially alleviated by the ability of cells to communicate with each other. In this way, a single cell which takes up the therapeutic gene can pass on the beneficial effects to its neighbouring cells. A similar "bystander effect" has been shown in model systems to be useful for the transfer of toxic metabolites between cancer cells after delivery of genes encoding enzyme prodrug activation systems. Another promising approach uses viral vectors which are capable of self-replication, ensuring more efficient delivery to a higher proportion of the target cells. In this case, specific safety features have to be built into these replication-competent viruses to ensure replication only within tumour cells. Clinical trials of many of these therapies are presently being carried out in hospitals worldwide. There is little information on long-term sequelae as most experiments are over short periods of time. There is a formal possibility of recombination, integration or transformation events with retroviral vectors but no evidence, so far, that this occurs.

### APPENDIX 6 – Ethical Review

**Extract (with permission) from a Home Office guidance document on: Local Ethical Review Processes**

© Cancer Research Campaign 1998 

'A local ethical review process could provide a mechanism to help certificate holders in meeting their responsibilities and could be the means of encouraging wider local involvement in addressing issues surrounding animal use. At the practical level, such consideration might involve asking whether the particular animal use proposed was appropriate and, if so, how the least animal suffering might be caused and welfare maximized.

The Home Secretary does not intend that any process over and above what is already required by the 1986 Act should be made mandatory. He invites you to consider, however, whether or not your own establishment would benefit from one or other of the local ethical review processes described above. The aim would be to maintain the awareness of all involved in laboratory animal care and use of their responsibilities towards their animal charges. Any of the ways described above would encourage this. Some of the processes will be more suitable for some institutions than others.'

### BIBLIOGRAPHY

### Official references

Animals (Scientific Procedures) Act (1986) HM Stationery Office, London, 1986

Guidance on the operation of the Animals (Scientific Procedures) Act 1986 HM Stationery Office, London (1990)

Code of practice for the housing and care of animals used in scientific procedures. HM Stationery Office, London (1989)

Code of practice for the housing and care of animals in designated breeding and supplying establishments. HM Stationery Office, London (1995)

### Key references

NB. This list includes both references cited in the text plus a number of other important references providing useful background information

Association of veterinary teachers and research workers (1989) Guidelines for the recognition and assessment of pain in animals. Universities Federation for Animal Welfare, Potters Bar, England

Balls M (1994) Replacement of animal procedures: alternatives in research, education and testing. Laboratory Animals 28: 193–211

Boven E, Winograd B (Eds.) The nude mouse in oncology research. CRC Press, Boca Raton, Ann Arbor, Boston, London (1991)

Denekamp J, Ed (1980) Quantitation of tumour response: A critical appraisal. Br. J. Cancer, 41 Suppl: IV, 1–331

Festing MFW (1994) Reduction of animal use: Experimental design and quality of experiments. Laboratory Animals, 28, 212–221

Flecknell PA (1994) Refinement of animal use – assessment and alleviation of pain and distress. Laboratory Animals 28: 222–231

Gay WI, Ed (1965) Methods of animal experimentation, Volume 1. Academic Press, New York

Geran RI, Greenberg NH, McDonald MM and Abbott BJ (1972) Evaluation of antileukemic agents in advanced leukemia L1210 in mice. Cancer Chemoth. Rep. Pt 3, 3: 1–103

Guidelines for the welfare of animals in rodent protection tests. A report from the Rodent Protection Test Working Party. Laboratory Animals (1994) 28: 13–18

Kallman RF, Ed (1987) Rodent tumor models. Pergamon Press, New York

Kallman RF, Brown JM, Denekamp J, Hill RP and Kummermehr J, Trott KR (1985). The use of rodent tumors in experimental cancer therapy. Cancer Res. 45: 6541–6545

Royal Society/Universities Federation for Animal Welfare (UFAW) (1987). Guidelines on the care of laboratory animals and their use for scientific purposes, Part 1 – Housing and care, Royal Society and UFAW, London

Roush W (1996) Hunting for animal alternatives. Science 274: 168–171

Russell WMS and Burch RL (1959) The principles of humane experimental technique. (reprinted 1992) Universities Federation for Animal Welfare London

Tuffery AA, Ed (1987) Laboratory Animals: An introduction for new experimenters. Wiley, Chichester (reprinted 1995)

UFAW (1987) The UFAW Handbook on the care and management of laboratory animals (ed. Poole TB). 6th edn. Longman Group UK Ltd, Harlow

Wallace J, Sanford J, Smith MW and Spencer KV (1990). The assessment and control of the severity of scientific procedures on laboratory animals. Report of the Laboratory Animal Science Association Working Party. Laboratory Animals 24: 97–130

### Other references

Bibby MC, Double JA, Ali SA, Fearon KC, Brennan RA and Tisdale MJ(1987) Characterisation of transplantable adenocarcinoma of the mouse colon producing cachexia in recipient animals. J. Natl. Cancer Inst. 78: 539–546

Boyd MR (1986) National Cancer Institute drug discovery and development. In Frei E, Freireich EJ (eds). Accomplishments in Oncology. Lippincot, Philadelphia. 67–76

Burtles SS, Newell DR, Henrar REC and Connors TA (1995). Revisions of general guidelines for the preclinical toxicology of new cytotoxic anticancer agents in Europe. Eur. J. Cancer 31A: 408–410

Donehower LA, Harvey M, Slagle BL, McArthur MJ, Montgomery CA, Butel JS and Bradley A (1992) Mice deficient for p53 are developmentally normal but susceptible to spontaneous tumours. Nature 356: 215–221

Fowler ME (1978) Restraint and handling of wild and domestic animals. Iowa State University Press, Ames

Hermanek PJ and Giedl J (1984) The adenoma–carcinoma sequence in AMMN-induced colonic tumours of the rat. Pathol. Res. Prac. 178: 548–554

Hollingshead MG, Alley MC, Camalier RF, Abbott RJ, Mayo JG, Malspeis L and Grever MR (1995) In vito cultivation of tumour cells in hollow fibers. Life Sciences 57: 131–141

Huggins C (1959) Chemically DMBA induced rat mammary tumours. J Exp.Med. 109: 25–41

Kerr DJ and Workman P (1994) New molecular target for cancer chemotherapy. CRC Press, Boca Raton

Kline I, Gang M, Tyrer DD, Vendetti JM, Artis EW and Goldin A (1972) Evaluation of antileukemic agents in advanced leukemia L1210 in Mice X. Cancer Chemoth. Rep. Pt 2, 3, 1–70

Kripke ML, Gruys E and Fidler IJ (1978) Metastatic heterogeneity of cells from an ultraviolet light induced murine fibrosarcoma of recent origin. Cancer Res. 38: 2962–2967

Mahon KA, Chepelinsky AB, Khillan JS, Overbeek PA, Piatigorsky J and Westphal H (1987) Oncogenesis of the lens in transgenic mice. Science 235: 1622–1628

Martin DS, Balis ME, Fisher B, Frei E, Freireich EJ, Heppner GH, Holland JF, Houghton JA, Houghton PJ and Johnson RK (1986) Role of murine tumour models in cancer treatment research. Cancer Res. 46: 2189–2192

Merz R, Wagner I, Habs M, Schmahl D, Amberger H and Bachmann (1981) Endoscopic diagnosis of chemically-induced autochthonous colonic tumors in rats. Hepatogastricenterol. 28: 53–57

Montgomery CA (1990) Oncologic and toxicologic research: alleviation and control of pain and distress in laboratory animals. The Cancer Bulletin 42: 230–237

Morton DB and Griffiths PHM (1985) Guidelines on the recognition of pain, distress and discomfort in experimental animals and an hypothesis for assessment. The Veterinary Record 116: 431–436

Moser AR, Pitot H C and Dove WF (1990) A dominant mutation that predisposes to multiple intestinal neoplasia in the mouse. Science 247: 322–324

Nordsmark M, Maxwell RJ, Wood PJ, Stratford IJ, Adams GE, Overgaard J and Horsman MR (1996) Effect of hydralazine in spontaneous tumours assessed by oxygen electrodes and P-31 magnetic resonance spectroscopy Br. J. Cancer 74: Suppl XXVII S232–S235

Redgate ES, Deutsch M and Boggs SS (1991) Time of death of CNS tumour-bearing rats can be reliably predicted by body weight-loss patterns. Laboratory Animal Science 41: 269–273

Twentyman PR, Brown, JM, Gray JW, Franko AJ, Scoles MA and Kallman RF (1980) A new mouse tumour model system (RIF-1) for comparison of end-point studies. J. Natl. Cancer Inst. 64: 595–604

Wood PJ, Stratford IJ, Sansom JM, Cattanach BM, Quinney RM and Adams GE (1992) The response of spontaneous and transplantable murine tumours to vasoactive agents measured by P-31 magnetic resonance spectroscopy. Int. J. Radiat. Oncol. Biol. Phys. 22: 473–476

*© Cancer Research Campaign 1998*